# Catadioptric Optical System Design of 15-Magnitude Star Sensor with Large Entrance Pupil Diameter

**DOI:** 10.3390/s20195501

**Published:** 2020-09-25

**Authors:** Yang Bai, Jianlin Li, Rongwei Zha, Ying Wang, Guangzhi Lei

**Affiliations:** 1 Institute of Photonics and Photon-Technology, National Key Laboratory of Photoelectric Technology and Functional Materials (Culture Base), Northwest University, Xi’an 710127, China; by@nwu.edu.cn (Y.B.); lijianlin@stumail.nwu.edu.cn (J.L.); zharongwei@stumail.nwu.edu.cn (R.Z.); wangyi2@stumail.nwu.edu.cn (Y.W.); 2 Space Optical Technology Research Department, Xi’an Institute of Optics and Precision Mechanics, Chinese Academy of Sciences, Xi’an 710119, China

**Keywords:** star sensor, catadioptric optical system, COS, large entrance pupil diameter, diffuse spot, 15-magnitude

## Abstract

The optical system is one of the core components for star sensors, whose imaging quality directly influences the performance of star sensors for star detection, thereby determining the attitude control accuracy of spacecrafts. Here, we report a new type of optical system with a catadioptric structure and a large entrance pupil diameter for a 15-magnitude star sensor. It consists of an improved Cassegrain system (R-C system), an aperture correction spherical lens group and a field of view correction spherical lens group. By embedding the secondary mirror of the R-C system into the output surface of the negative spherical lens of the aperture correction spherical lens group, the blocking of incident light is eliminated from the secondary mirror holder. After the structure optimization, the catadioptric optical system (COS) had a spectral range of 450 nm–950 nm, an entrance pupil diameter of 250 mm, a half-diagonal field of view of 1.4° and a focal length of 390 mm. By using theoretical calculations and experimental measurements, it was verified that the COS, with the ability to correct astigmatism, lateral color and distortion, can fulfill the detection of 15-magnitude dark stars.

## 1. Introduction

The optical system is one of the core components of star sensors, and its imaging quality directly affects the detection ability of the star sensors for star detection, which in turn affects the attitude control accuracy of the aerospace vehicle [1,2,3,4,5]. To improve the detection ability of the star sensor, the optical systems should have a larger entrance pupil diameter and a wider spectral range, and the distortion and chromatic aberration generated during the imaging process should be minimized. Optical systems of the star sensors have been extensively studied since the 1950s, of which most are designed with refraction and reflection structures [6,7,8,9]. The refractive optical systems are easy to realize high-quality large-field imaging, but they also have inherent defects such as a small aperture, a lot of optical lenses and it is difficult to correct their secondary spectrum [9,10,11]. The reflective optical systems have the advantages of a large system aperture, a small number of optical lenses and no chromatic aberration. However, there are some problems in its structure, such as a low energy utilization rate of space light, weak aberration correction ability of the edge of the field of view and a lot of challenges in its structure design [12,13]. In recent years, researchers have proposed a catadioptric optical system (COS) which combines a Schwarzschild system with an aspheric compensation lens group, or a Cassegrain system (R-C system) with a spherical compensation lens group [14,15,16]. The primary mirror of the former catadioptric structure is convex aspherical and its aperture is small, resulting in a small entrance pupil diameter of the COS. Compared with the entrance pupil size of the transmission optical system, there is no obvious advantage. Besides, the distortion of the edge of the field of view of the system is too large (close to 2%), and the processing of the aspheric compensation lens groups is also difficult. The mirror of the latter catadioptric structure will neither produce chromatic aberration nor affect the displacement of the image plane. The spherical aberration and coma can be corrected by the primary and secondary lenses which are both hyperbolic. The spherical compensation lens group can correct residual astigmatism, field curvature and distortion [17]. More importantly, a star sensor with this structure can obtain a higher star detection ability because of its larger entrance pupil diameter [16,18]. However, the bracket of the mirror further aggravates the central blockage of incident light and causes a greater loss of light energy. Therefore, the detection capability of the star sensors with this COS is difficult to improve further.

In this article, we demonstrate a new large aperture COS for a 15-magnitude star sensor that combines an R-C system, an aperture correction spherical lens group and a field of view correction spherical lens group. The secondary mirror of the R-C system was embedded on the output surface of the negative spherical lens of the aperture correction spherical lens group, which was used to avoid the blocking of the incident light by the bracket of the secondary mirror. It was operated at an entrance pupil diameter of 250 mm, a half-diagonal field of view of 1.4°, a focal length of 390 mm and in a spectral range of 450–950 nm. The results of the image quality evaluation showed that the COS had a better ability to correct various aberrations. Through theoretical simulation and experimental measurement, respectively, it was verified that the diffusion spot obtained by the COS prototype was within the pixel size of 14 × 14 μm. Our results show the feasibility and practicability of using this large aperture COS to detect 15-magnitude dark stars in a wide spectrum.

## 2. Main Technical Parameters of the Optical System

According to the application requirements of a certain aerospace vehicle, the main functional indicators such as the spectral range, limiting magnitude, field of view and angular resolution of a star sensor with 15 limit star detection capabilities are listed in this paper, as shown in Table 1:

The star sensor requires its optical system to detect stars of the 15-magnitude in the 450–950 nm spectral range and uses a Charge-coupled Device (CCD) detector with corresponding pixels to image the detected stars. The ultimate goal of imaging is to obtain a diffuse spot with a certain size on a CCD detector in the full field of view, whereas the energy distribution of the diffuse spot is close to the Gaussian distribution in order to calculate its centroid [19,20]. Therefore, the CCD detector should be determined before designing the optical system. The choice of a CCD detector can be determined by the equation of the optical system’s *FOV* [21], which is defined as:

(1) FOV=n·α

where *FOV* = 2° and *α* = 33.5 μrad are the field of view and angular resolution of the optical system, respectively. *n* is the number of pixels, which is calculated to be 1024 for the CCD detector. Therefore, a back-illuminated scientific CCD detector (e2v technologies Inc., Grenoble, France, CCD47-10) with 1024 × 1024 pixels is selected in this paper and its specific parameters are summarized in Table 2.

The focal length of the optical system can be calculated according to Equation (2) [22]:

(2) $$f=\frac{b}{\alpha }$$

where *f* and *α* are the focal length and angular resolution of the optical system, respectively. In this case, *b* = 13.3 μm was the single pixel size of the CCD detector. From the data in Table 1 and Table 2, *f* was calculated as 388.06 mm, so the focal length of the optical system was determined to be 390 mm.

In the process of optical design, the diagonal field of view (*ω*) is usually employed to represent the field of view of the optical system. The relationship between the field of view and the focal length of the optical system is governed by Equation (3) [10]:

(3) $$tg(\omega )=\frac{\sqrt{H^{2}+V^{2}}}{2f}$$

where *ω* and *f* are the half-field of view and the focal length of the optical system, respectively. *H* and *V* represent the overall length and width of the CCD pixel, respectively. According to Equation (3), *ω* was set at 1.4°.

As a parameter to measure the ability to detect the weakest magnitude, the detection sensitivity of the star sensor determines the number of stars in its field of view. The detection sensitivity model of the star sensor establishes the relationship with the detected magnitude by calculating the signal-to-noise ratio (SNR) of the starlight signal, which is expressed as follows [23]:

(4) $$SNR=\frac{S}{\sqrt{S+B+N_{sensor}^{2}}}\geq V_{th}$$

where *S*, *B* and *N_sensor_* are the electron number of the target signal, the electronic number of the background noise and the electronic number of the CCD detector noise, respectively; *V_th_* is the threshold of signal-to-noise ratio, which satisfies certain detection rates and false alarm rates. Considering that the star sensor works in space, Equation (4) can be rewritten as follows if it omits the background noise:

(5) $$S\geq \frac{V_{th}^{2}+\sqrt{V_{th}^{4}+4V_{th}^{2}N_{sensor}^{2}}}{2}$$

When using the CCD detector with a single pixel area of 13.3 × 13.3 μm, the calculation equation of the electron number of the target signal can be expressed as:

(6) $$S=\frac{\pi }{N_{spread}}{\left(\frac{D}{2}\right)}^{2}T\Delta \lambda \varphi_{m}Q_{E}\tau$$

where *D*, *T* and Δ*λ* are the entrance pupil diameter, transmittance and spectral range of the optical system, respectively; *φ_m_* is the luminous flux of stars of m-magnitude outside the earth’s atmosphere and *φ_m_* = 10^(15 − 2 × m)/5^; *τ* is the integration time; *N_spread_* = 13.3 × 13.3 μm is the pixel area of the diffuse spot distribution; *Q_E_* is the weighted average of the quantum efficiency of the CCD detector. It should be noted that the quantum efficiency of the CCD detector exceeds 90% in the spectral range from 450 nm to 850 nm. From 850 nm to 950 nm, its quantum efficiency is higher than 50%. Therefore, the *Q_E_* value provided by the manufacturer is around 90%, which is calculated based on the photon number distribution in the spectral range from 450 nm to 950 nm.

A mathematical model of the magnitude sensitivity of the star sensor could be obtained by using Equations (5) and (6):

(7) $$m\leq -2.5\mathrm{lg}\left(\frac{10^{-3}(V_{th}^{2}+\sqrt{V_{th}^{4}+4V_{th}^{2}N_{sensor}^{2}})N_{spread}}{2\pi {\left(\frac{D}{2}\right)}^{2}T\Delta \lambda Q_{E}\tau }\right)$$

Under a space background, in order to achieve the effective detection of dark stars with *m* = 15, the detection efficiency and false alarm rate of the star sensor are set to be greater than 99% and less than 1%, respectively. In order to satisfy these two conditions, *V_th_* is determined to be 5. In theory, each surface of the optical lens will be antireflection coated with a transmittance higher than 99%, and the reflective surfaces of the two mirrors will be antitransmission coated with a reflectivity higher than 99%. When the absorptivity of the optical glass, the number of optical lenses and the number of optical mirrors are set to 1%, 5 and 2, respectively, the theoretical transmittance of the optical system is *T* = 0.99^10^ × (1 − 0.01)^5^ × 0.99^2^ = 0.84. Since the absorption coefficient of the optical glass fluctuates in the range from 450 nm to 950 nm, the initial absorption coefficient of the optical system is determined to be 80%, provided that the blocking ratio generated by the optical mirror is not considered. When the relevant parameters in Table 2 were substituted into Equation (7), the theoretical value of the entrance pupil diameter *D* of the optical system should not have been less than 207.4 mm when *τ* was 0.1 s. When the shading ratio by the optical mirror was set to 55%, the *D* was calculated to be 248.35 mm. In order to facilitate the processing of optical lenses, the design value of the *D* was determined to be 250 mm.

Considering the technical requirement that the single-star measurement accuracy of the star sensor is less than 1″, the centroid localization accuracy of the optical system can be obtained by using Equation (8) of single-star measurement accuracy [24]:

(8) $$\zeta_{s}=\frac{A_{FOV}}{N_{pixel}}\sigma_{c}$$

where *ζ_s_*, *A_FOV_*, *N_pixel_* and *σ_c_* are the single-star measurement accuracy, the field of view, the number of pixels and the centroid localization accuracy, respectively. When *ζ_s_* < 1″, *A_FOV_* = 2° and *N_pixel_* = 1024, then *σ_c_* < 0.142 pixels.

In general, the average angle error of the measurement obtained, based on research experience, does not exceed 10″, which corresponds to the error of the centroid localization accuracy caused by the relative distortion of the optical system. Therefore, the maximum relative distortion of the optical system imaging can be obtained as follows:

(9) $$\frac{q\cdot h}{f}\leq \frac{10}{3600}\times \frac{\pi }{180}$$

where *q* is the relative distortion of the optical system imaging, *f* is the focal length of the optical system, *h* = *f* × *tg*(*ω*) is the height of the half-field of view on the ideal image plane, and *ω* is the half-field of view. When *f* = 390 mm and *ω* = 1.4°, *q* < 0.2%.

In general, the center of the diffusion spot cannot exactly coincide with the center of a pixel. When the size of the dispersion spot matched that of a pixel, more than 80% of the diffuse spot energy distribution was required to be within a range of 2 × 2 pixels or 26.6 × 26.6 μm, corresponding to a pixel size of 13.3 × 13.3 μm. In addition, under the premise of a 450–950 nm wavelength range and a 600 nm center wavelength, the magnification chromatic aberration of the optical system designed, based on the Rayleigh scattering theory, should be as close as possible to the size of the Airy spot, i.e., the lateral color was determined to be less than 2 μm.

In summary, the main design specifications of the optical system are shown in Table 3.

## 3. Structure Design of the Optical System

On the premise of complying with the technical requirements of the optical system in Table 3, a new COS for the 15-magnitude star sensor was proposed. It was composed of an R-C system, an aperture correction spherical lens group and a field of view correction spherical lens group. The purpose of this design was to improve the measurement accuracy of the star sensor and increase the entrance pupil diameter of the COS while eliminating aberrations in the wide spectral range of 450–950 nm [25]. Firstly, in the design process of the optical system, the initial structure size of the R-C system in the COS was determined based on the primary aberration theory and the equation of a coaxial two mirror type coefficient [26,27], so that the position between its primary mirror and the secondary mirror was fixed. Subsequently, the spherical lens groups were added gradually and the structure of the COS was optimized by Zemax software, so as to increase the field of view and correct the aberrations of the whole COS to a minimum. The initial pupil diameter of the two mirrors of the R-C and the aberrations of the whole COS were calculated as follows:

(10) $$D_{2}=\frac{\alpha \beta }{1+\beta }D_{1}$$

(11) $$S_{1}=\frac{\alpha {\left(\beta -1\right)}^{2}(\beta +1)-\alpha {\left(\beta +1\right)}^{3}e_{2}^{2}-\beta^{3}(1-e_{1}^{2})}{4}$$

(12) $$S_{2}=\frac{\left(1-\alpha \right)\left[\alpha {\left(\beta +1\right)}^{3}e_{2}^{2}-\alpha {\left(\beta -1\right)}^{2}(\beta +1)\right]}{4\alpha \beta }-\frac{1}{2}$$

(13) $$S_{3}=\frac{{\left(1-\alpha \right)}^{2}\left[\alpha {\left(\beta -1\right)}^{3}(\beta +1)-\alpha {\left(\beta +1\right)}^{3}e_{2}^{2}\right]}{4\alpha^{2}\beta^{2}}-\frac{\left(1-\alpha )(\beta +1)(\beta -1\right)}{\alpha \beta }-\frac{\beta (\alpha -1)-1}{\alpha }$$

(14) $$S_{4}=\beta -\frac{1+\beta }{\alpha }$$

(15) $$S_{5}=\frac{{\left(1-\alpha \right)}^{3}\left[\alpha {\left(\beta +1\right)}^{3}e_{2}^{2}-\alpha {\left(\beta -1\right)}^{2}(\beta +1)\right]}{4\alpha^{3}\beta^{3}}-\frac{3{\left(1-\alpha \right)}^{2}(1+\beta )(1-\beta )}{2\alpha^{2}\beta^{2}}-\frac{2(1-\alpha )(1+\beta )}{\alpha^{2}\beta }$$

where *D*_1_ = 250 mm is the initial pupil diameter of the primary mirror, *D*_2_ is the initial pupil diameter of the secondary mirror and *α* = *D*_2_/*D*_1_ and *β* are the obstruction ratio and magnification of the secondary mirror, respectively. Considering that the bracket of the secondary mirror blocked the incident light, the maximum initial value of *D*_2_ was determined to be 107.5 mm, and *α* did not exceed 0.43; *S*_1_, *S*_2_, *S*_3_, *S*_4_ and *S*_5_ are the spherical aberration, coma, astigmatism, distortion and field curvature, respectively; *e*_1_^2^ and *e*_2_^2^ are the surface parameters of the primary and secondary mirrors, respectively, which are related to the machining tolerance of the primary and secondary mirrors.

In an R-C system, the primary lens and the secondary lens both have a hyperboloid surface, which can effectively correct the primary spherical aberration and coma, considering that *S*_1_ = *S*_2_ = 0. From the above Equations (10)–(15), the following equations were obtained:

(16) $$e_{1}^{2}=1+\frac{2\alpha }{(1-\alpha )\beta^{2}}$$

(17) $$e_{2}^{2}=\frac{2\beta +(1-\alpha )(1+\beta ){\left(1-\beta \right)}^{2}}{(1-\alpha ){\left(1+\beta \right)}^{3}}$$

**Figure 1:**
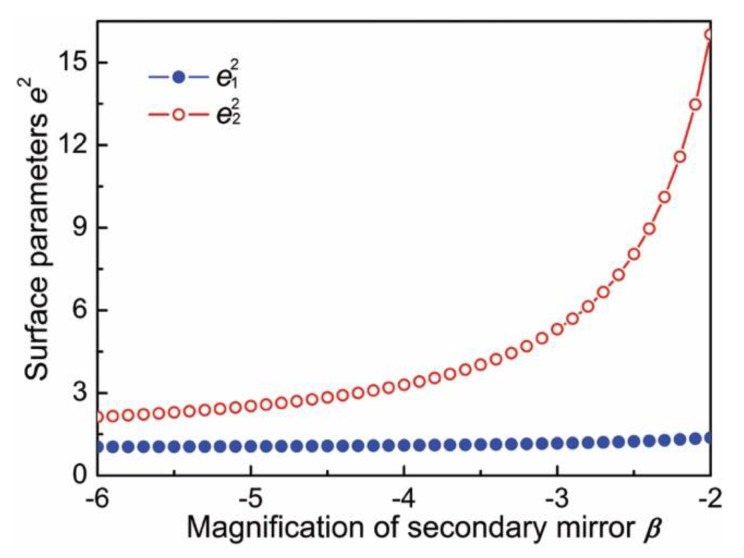
Curves of *e*_1_^2^ and *e*_2_^2^ versus *β* value.

When *α*_max_ = 0.43 was substituted in Equations (16) and (17), the variation of *e*_1_^2^ and *e*_2_^2^ versus *β* value was simulated and shown in Figure 1. The results showed that, *e*_2_^2^ changed rapidly and *e*_1_^2^ changed slowly with *β*. This shows that the *β* value had little influence on the processing tolerance of the primary mirror, but presented a stronger impact on the processing tolerance of the secondary mirror. The smaller *e*_2_^2^ value corresponded to the tighter machining tolerance of the secondary mirror. Therefore, *β* value was finally determined as −5 based on the comprehensive consideration of tolerance requirements, design difficulty and design experience.

**Figure 2:**
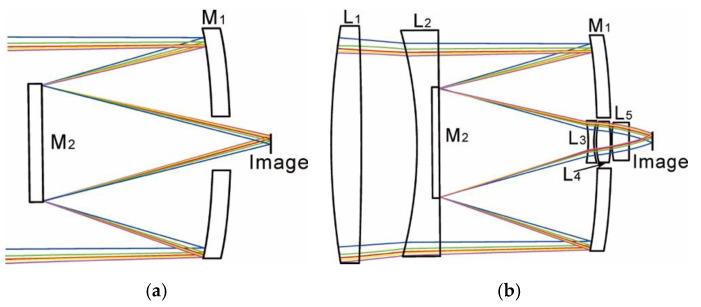
Schematic diagrams of (**a**) initial optical system structure, (**b**) optimized optical system structure.

After substituting *α*_max_ = 0.43 and *β* = −5 into Equations (16) and (17), the solution was *e*_1_^2^ = 1.0604 and *e*_2_^2^ = 2.5241. The surface shapes of the primary and secondary mirrors for the R-C system were then determined as hyperboloid surfaces. When the initial distance *l* between the primary mirror and the secondary mirror was set as 140 mm, the focal point of the R-C system was located near the image plane. The initial structure of the R-C system is shown in Figure 2a. However, the number of variable parameters of the R-C system with a larger entrance pupil diameter of 250 mm was very limited, so other aberrations were inevitably introduced and they could not be corrected well [25,26,27]. As shown in Figure 2b, the primary mirror M_1_ and the secondary mirror M_2_ constituting the R-C unit in the COS is a hollow hyperbolic mirror and a solid hyperbolic mirror, respectively. The spherical aberration and coma can be corrected by the mirrors M_1_ and M_2_. Adding an aperture correction spherical lens group (L_1_, L_2_) in front of the mirror M_2_ corrected residual astigmatism, field curvature and distortion caused by the 1.4° half-diagonal field of view of the COS. Concurrently, the mirror M_2_ was embedded on the output surface of the negative spherical lens L_2_ for the aperture correction spherical lens group, which was used to eliminate the blocking of the incident light by the bracket of the secondary mirror. In addition, a field of view correction spherical lens group (L_3_, L_4_, L_5_) added between the secondary mirror M_2_ and the image plane not only expanded the field of view of the COS, but also used a combination of positive and negative lenses to reduce the chromatic aberration introduced by each spherical lens.

Finally, the initial structural parameters of the R-C system of *D*_1_ = 250 mm, *D*_2_ = 107.5 mm, *e*_1_^2^ = 1.0604, *e*_2_^2^ = 2.5241 and *l* = 140 mm were input into the optical design software Zemax. Using the operand control in the Zemax software, the structure of the COS was optimized to finally meet the imaging requirements of the 15-magnitude star sensor [28]. Among them, the operand EFFL was used to control the total focal length of the COS to reach 390 mm. The TTHI operand was used to control the radius of curvature (*r*_ij_, i: left, j: right), thickness (*t*) and air interval (*l*) of five lenses from L_1_ to L_5_ and two mirrors, M_1_ and M_2_. The DMVA operand was used to control the diameter of each lens and mirror in the COS. The AXCL operand was used to control the lateral color of the COS. The hollow diameter of the mirror M_1_ was the same as that of the three lenses L_3_, L_4_ and L_5_. The exit surface of the lens L_2_ and the reflective surface of the mirror M_2_ had the same radius of curvature, and the mosaic depth of the mirror M_2_ in the lens L_2_ was the same as its thickness. The optimized structure parameters of the COS for the 15-magnitude star sensor are shown in Table 4.

**Figure 3:**
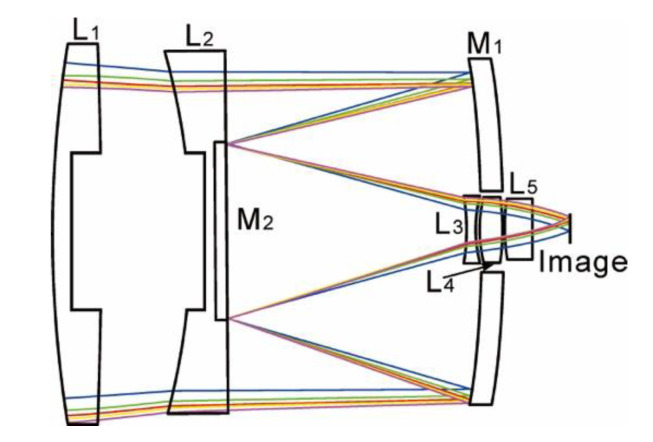
Schematic diagrams of optical system structure after mass reduction.

With the characteristics from Table 4, the mass of the lenses L_1_ and L_2_ exceeded 6 kg. In order to reduce the mass, the structures of the lenses L_1_ and L_2_ needed to be modified, as shown in Figure 3. On the premise of ensuring the structural strength, a cylinder with a depth of 22 mm and a diameter of 130 mm was removed from the exit surface of the lens L_1_. A cylinder with a depth of 15 mm and a diameter of 130 mm was removed from the incident surface of the lens L_2_. After the improvement of the above structure, the mass of the optical system was reduced by about 1.1 kg.

## 4. Image Quality Evaluation

Normally, the spot diagram, distortion curve, encircled energy curves, lateral aberration curves etc. are used to evaluate the imaging quality of the optical system for the star sensor.

**Figure 4:**
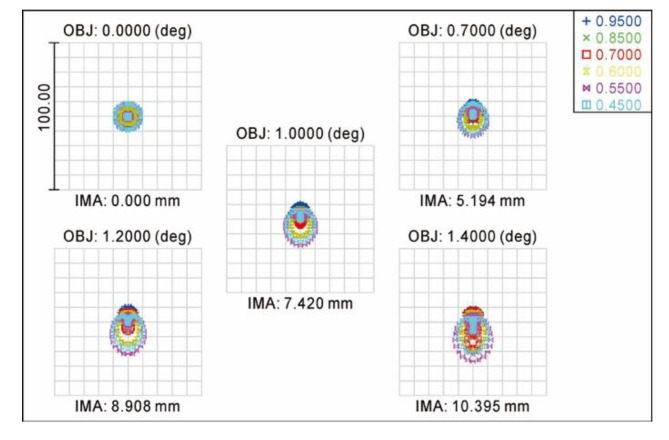
Spot diagram of diffuse spots.

In the spectral range of 450–950 nm, Figure 4 shows the spot diagrams of the diffuse spots from the COS under five half-diagonal field of views of 0.0°, 0.7°, 1.0°, 1.2° and 1.4°. Table 5 shows the radii and centroid localization accuracies of the diffuse spots under the different half-diagonal field of views. It was clearly seen that the distributions of the diffuse spots were not only basically round and concentrated, but also had a certain degree of dispersion and a good symmetry. The radius of the diffuse spot increased with the field of view. Under the 1.4° half-diagonal field of view, the radius of the diffuse spot reached a maximum of 7.515 μm. The centroid localization accuracy reached the maximum value of 0.0026 pixels when the half-diagonal field of view was 0.7°, which fully met the design specification of the COS where the centroid localization accuracy of the diffuse spot needed to be less than 0.142 pixels.

**Figure 5:**
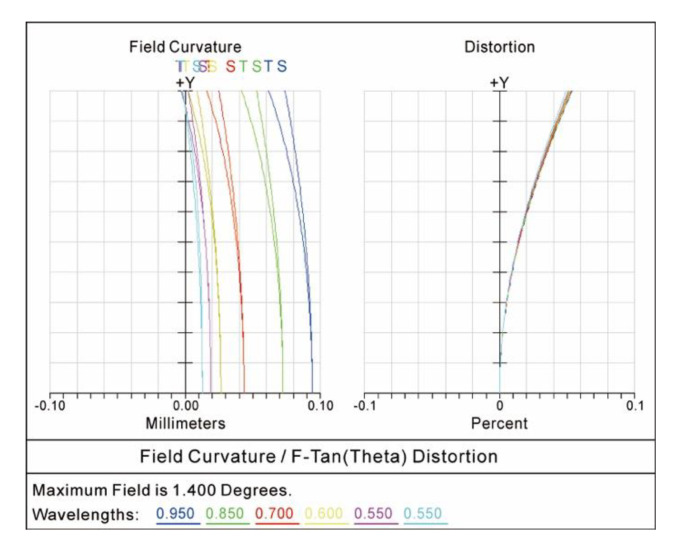
Relative distortion curves.

For the optical system of the star sensor, the smaller relative distortion in the imaging diagram was necessary for the star sensor to obtain a higher measurement accuracy. The relative distortion curve of the COS is shown in Figure 5. The maximum relative distortion was 0.081% in the whole diagonal field of view from −1.4° to +1.4°, which fully met the design specification of the relative distortion of less than 0.1% in the spectral range of 450–950 nm.

**Figure 6:**
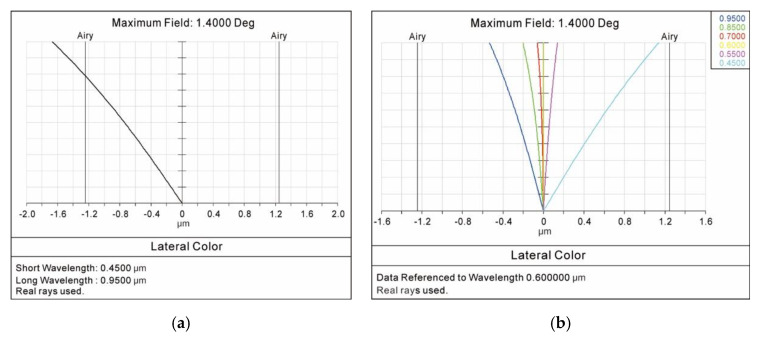
Lateral color curves (**a**) between the short wavelength of 450 nm and the long wavelength of 950 nm, (**b**) between different wavelengths (450–950 nm) and the center wavelength of 600 nm.

In the whole diagonal field of view from −1.4° to +1.4°, Figure 6a shows the lateral color curve between the short wavelength of 450 nm and the long wavelength of 950 nm. Figure 6b shows the lateral color curves between different wavelengths (450–950 nm) and the center wavelength of 600 nm. The results showed that the maximum of the lateral color was −1.67 μm. The design specification of the lateral color that was less than 2 μm was fully demonstrated.

**Figure 7:**
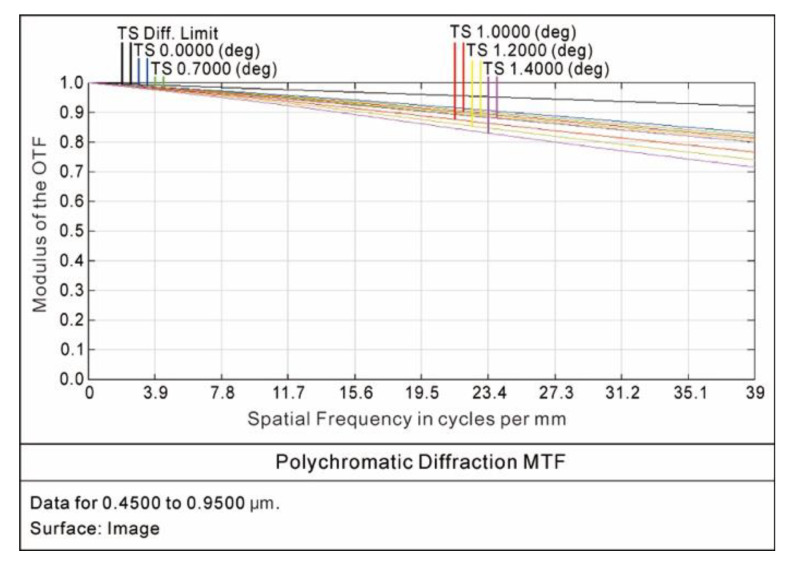
MTF curves.

**Figure 8:**
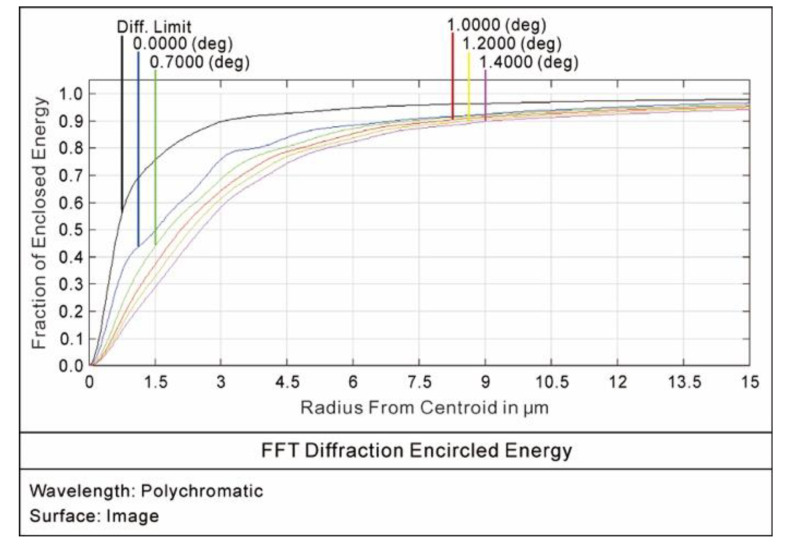
Encircled energy curves.

The curves of the optical modulation transfer function (MTF) are shown in Figure 7. The modulus of the MTF under the different fields of view at the Nyquist frequency of 39 lp/mm was greater than 0.72, meeting the design specification of the MTF, which was greater than 0.5. The curves of the encircled energy under the different fields of view are shown in Figure 8. The energy distributions of the diffuse spots were close to the Gaussian normal distribution. A total of 80% of the energy and 95% of the energy contained in the diffuse spots were distributed within a radius of 5.2 μm and a radius of 11.3 μm, respectively. The simulation results met the design requirement that the 80% energy of the diffuse spot was distributed at 26.6 × 26.6 μm.

## 5. Measurement of the Diffuse Spot

**Figure 9:**
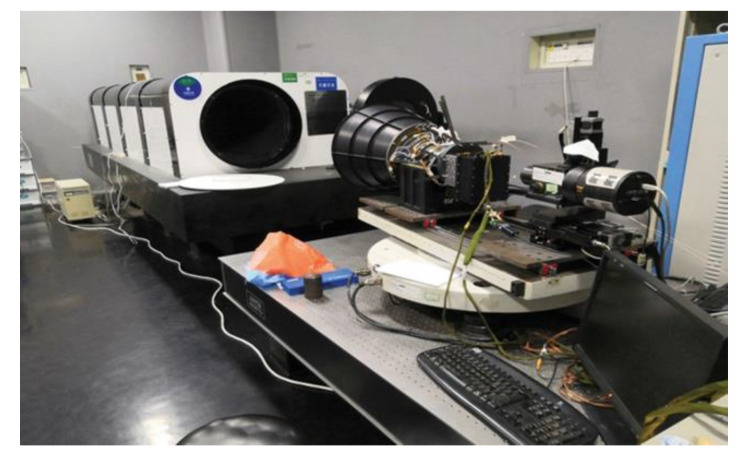
Experimental measurement device used to measure the size of diffuse spot.

During the design of our optical system, errors caused by unfavorable factors such as processing, installation, adjustment of the optical devices and the external environment could not be considered in the simulation of the diffuse spot size. Therefore, under different defocus conditions, the experimental measurement images of the diffuse spots were obtained from the COS prototype, and the diffuse spot sizes were quantitatively analyzed. Such work was very necessary to more accurately evaluate the imaging quality of the star sensor. The experimental measurement device used for the diffuse spot size is shown in Figure 9. A xenon lamp with an emission spectrum (190–1100 nm) was used as the light source of a target star simulator. An integrating sphere with a star point (0.01 mm) and collimator were used to simulate stars at infinity. After the parallel light emitted by the target star simulator passed through the COS prototype and was magnified by a 50× microscope, a CCD detector (CCD47-10) imaged the diffuse spot near its focal plane to obtain the experimental measurement image. When the COS prototype and the CCD detector were placed on an electro-control turntable at the same time, the diffuse spots were measured in different fields of view. The positioning accuracy and reset accuracy of the electro-control turntable were more than 1.5” and 1”, respectively, and the rotation angle range was 0–360°.

In order to simplify the image processing algorithm, the image noises of the diffuse spots were regarded as background noise and removed by Gaussian filtering. In the image processing process, the diffuse spot was regarded as a random light spot that obeyed the Gaussian distribution. The specific step of filtering involves initially substituting the distance from other pixels in the neighborhood to the center of the neighborhood as a parameter into the two-dimensional Gaussian function to calculate a 3 × 3 Gaussian template. The two-dimensional Gaussian function is calculated as follows:

(18) $$G(x,y)=\frac{1}{2\pi \sigma^{2}}e^{\frac{-(x^{2}+y^{2})}{2\sigma^{2}}}$$

where (*x*, *y*) are point coordinates, which can be considered as integers in image processing. The standard deviation is *σ*.

**Figure 10:**
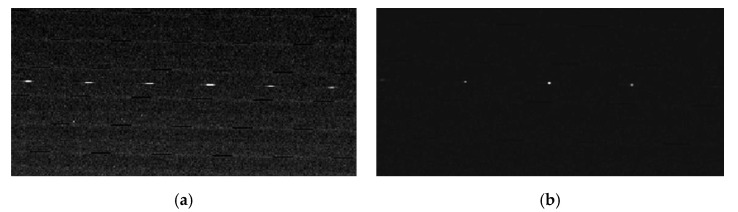
Images of diffuse spot collected by the COS prototype (**a**) before noise reduction, (**b**) after noise reduction.

**Figure 11:**
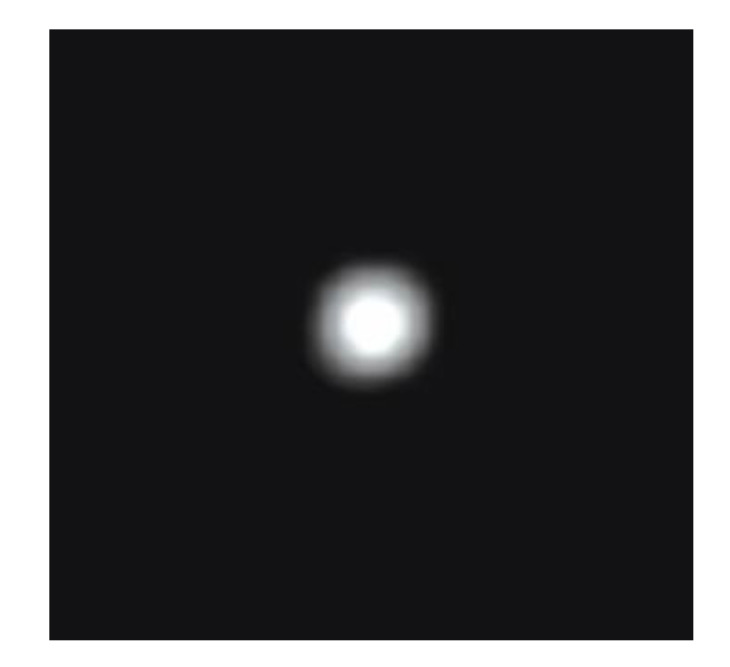
Optical speckle test image.

If the template is in decimal form, it needs to be normalized and the value of the upper left corner of the template is normalized to 1. Then, the center of the Gaussian template needs to align with the image matrix in order to be processed, then multiplied with the corresponding elements and added together. Finally, set “0” where there is no element. Each element is calculated separately, and the output matrix obtained is the result of Gaussian filtering. The images of the diffuse spot collected by the COS prototype before and after noise reduction are shown in Figure 10a,b, respectively. Gaussian filtering is a frequency domain processing method. When analyzing the frequency characteristics of an image signal, the edges, jumps and grain noise of the image usually represent the high-frequency components of the image signal, while the large background area represents the low-frequency components of the image signal. Filtering the high-frequency components can remove the background noise, so that the image is smooth. Therefore, it can be seen from Figure 10a,b that the images of the diffuse spots before filtering looked like ellipses and then became circles. Figure 11 shows the image of the diffuse spot from the COS prototype measured at a defocus amount of 0.02 mm and a field of view of 0°. The strong amount of roundness of the diffuse spot indicated that the COS prototype designed for the 15-magnitude star sensor was less affected by distortion and color aberration.

**Figure 12:**
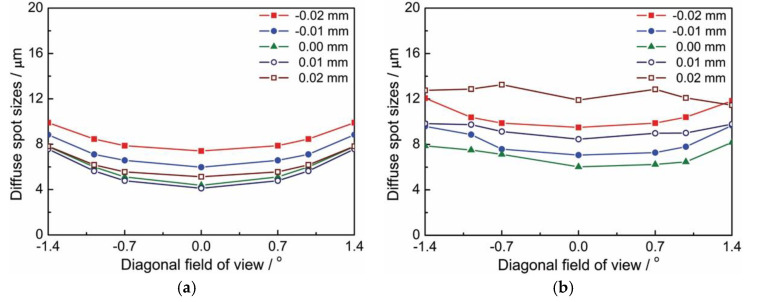
Diffuse spot sizes of the COS prototype by (**a**) theoretical calculations, (**b**) experimental measurements.

The theoretical value of the diffuse spot size of the COS was obtained using the Zemax software, under different defocus amounts and fields of view, as shown in Figure 12a. A centroid algorithm based on the gray distribution was used to perform the image processing of the diffuse spots after noise reduction, from which the experimental measurement values of the diffuse spots were obtained under different defocus amounts and different fields of view, as shown in Figure 12b. Comparing Figure 12a,b, one can easily find that the theoretical value of the diffuse spot size obtained by the COS prototype was basically consistent with the experimental measured value, and both were less than 14 μm. This means the diffuse spots were distributed in 14 × 14 μm within. Both theoretical and experimental results showed that the structural design of the COS prototype met the index specifications of the 15-magnitude star sensor.

## 6. Conclusions

In conclusion, according to the technical requirements that the star sensor of a spacecraft should have 15-magnitude detection capability, the CCD detector type of a star sensor was determined, and the main functional indexes of the optical system, such as spectral range (450–950 nm), half-diagonal field of view (1.4°), focal length (390 mm) and entrance pupil diameter (250 mm), were obtained based on theoretical calculation. Based on this, a new type of COS, using a combination of an R-C system, an aperture correction spherical lens group and a field of view correction spherical lens group, was designed for a 15-magnitude star sensor. The image quality of the COS was further evaluated. The evaluation parameters verified that the COS had the capability to correct astigmatism, lateral color and distortion. For example, its secondary shielding ratio was 0.43, 80% diffuse spot energies were distributed within a radius of 5.2 μm, the maximum relative distortion was 0.081%, the modulus of the MTF at Nyquist frequency of 39 lp/mm was greater than 0.72 and the maximum of the lateral color was −1.67 μm. Through theoretical calculations and experimental measurements, it was proven that the sizes of the diffuse spots from the COS prototype under different defocus amounts and different fields of view were all distributed in an area within 14 × 14 μm. The results showed that our COS meets the technical specifications for detecting 15-magnitude dark stars and provides a promising reference for detecting dark stars with higher magnitudes.

## Figures and Tables

**Table 1:** Star sensor functional indexes.

Parameters	Values
Spectral range	450–950 nm
Center wavelength	600 nm
Limiting magnitude	15
Field of view	>2° × 2°
Angular resolution	>33.5 μrad
Total length	<900 mm
Detection efficiency	>99%
False alarm rate	<1%
Single-star measurement accuracy	<1″

**Table 2:** Main parameters of CCD.

Parameters	Values
Number of pixels	1024 × 1024
Spectral range	200–1100 nm
Pixel size	13.3 × 13.3 μm
Weighted average of quantum efficiency	90% from 450 nm to 950 nm
Readout noise	2.0 e^−^·rms at 20 kHz

**Table 3:** Main design specifications of the optical system.

Parameters	Values
Spectral range	450–950 nm
Center wavelength	600 nm
Focal length	390 mm
Entrance pupil diameter	250 mm
Half-field of view (diagonal)	1.4°
Centroid localization accuracy	<0.142 pixels
Relative distortion	<0.2%
Lateral color	<2 μm
Modulation transfer function	≥0.5@39 lp/mm
80% diffuse spot energy distribution	<26.6 μm

**Table 4:** Optimized parameters of the catadioptric optical system (COS).

Lens	Radius of Curvature (*r*_ij_/mm)	Thickness (*t*/mm)	Pupil Diameter (*d*/mm)	Air Thickness (*l*/mm)	Glass
L_1_	791.965	37.000	285.3	65.0 L_1_→L_2_	SILICA
	−4921.341				
L_2_	−659.988	28.000	269.3	189.0 L_2_→M_1_	SILICA
	−10500				
M_1_	−849.753	15.875	257.2	−189.0 M_1_→M_2_	Mirror
	−849.753				
M_2_	−10500	8.750	132.9	178.0 M_2_→L_3_	Mirror
	−10500				
L_3_	−146.253	7.000	49.5	1.9 L_3_→L_4_	H-LAF2
	107.419				
L_4_	107.982	17.500	48.4	0.6 L_4_→L_5_	H-LAK10
	−132.483				
L_5_	138.007	22.000	45.3	28.0 L_5_→Image	H-LAK10
	722.838				

**Table 5:** Radii and centroid localization accuracies of diffuse spots.

Field of View (°)	Radius (μm)	Centroid Localization Accuracy (Pixel)
0	4.027	0
0.7	4.982	0.0026
1.0	5.875	0.0018
1.2	6.629	0.0006
1.4	7.515	0.0001
